# Impact of Delay on Hospitalization in Older Patients With Head and Neck Cancer: A Multicenter Study

**DOI:** 10.1177/01945998211072828

**Published:** 2022-01-19

**Authors:** Rosanne C. Schoonbeek, Suzanne Festen, Roza Rashid, Boukje A.C. van Dijk, György B. Halmos, Lilly-Ann van der Velden

**Affiliations:** 1 Department of Otorhinolaryngology and Head and Neck Surgery, University Medical Center Groningen, University of Groningen, Groningen, the Netherlands; 2 University Center for Geriatric Medicine, University Medical Center Groningen, University of Groningen, Groningen, the Netherlands; 3 Department of Head and Neck Oncology and Surgery, Netherlands Cancer Institute/Antoni van Leeuwenhoek, Amsterdam, the Netherlands; 4 Department of Research, Netherlands Comprehensive Cancer Organisation, Utrecht, the Netherlands; 5 Department of Epidemiology, University Medical Center Groningen, University of Groningen, Groningen, the Netherlands

**Keywords:** head and neck neoplasms, treatment delay, hospitalization, time to treatment, overall survival

## Abstract

**Objective:**

To assess the impact of delay in treatment initiation on hospitalization, overall survival, and recurrence in older patients with head and neck cancer (HNC).

**Study Design:**

Retrospective multicenter study.

**Setting:**

Two tertiary referral centers.

**Methods:**

All patients with newly diagnosed HNC (≥60 years) treated between 2015 and 2017 were retrospectively included. Time-to-treatment intervals were assessed (ie, calendar days between first visit and start of treatment). Multiple multivariable models were performed with hospital admission days (>14 days), survival, and recurrence as dependent outcome variables.

**Results:**

In total, 525 patients were enrolled. The mean age was 70.7 years and 70.7% were male. Median time to treatment was 34.0 days, and 36.3% started treatment within 30 days (*P* = .576 between centers). Patients with radiotherapy had longer time to treatment than surgical patients (39.0 vs 29.0 days, *P* < .001). Current smoking status, stage IV tumors, and definitive radiotherapy were significantly associated with delay in the multivariable analysis. Time-to-treatment interval ≥30 days was a significant predictor of longer hospital admission (>14 days) in the first year after treatment in an adjusted model (odds ratio, 4.66 [95% CI, 2.59-8.37]; *P* < .001). Delay in treatment initiation was not associated with overall survival or tumor recurrence.

**Conclusion:**

This study highlights the importance and challenges of ensuring timely treatment initiation in older patients with HNC, as treatment delay was an independent predictor of hospitalization. During oncologic workup, taking time to consider patient-centered outcomes (including minimizing time spent in hospital) while ensuring timely start of treatment requires well-structured, fast-track care pathways.

As a result of today’s aging society, the proportion of older patients within the head and neck cancer (HNC) population is subsequently increasing.^1,2^ Treatment for patients with HNC often yields intensive multimodality treatment in an anatomically and functionally complex area, sometimes resulting in severe disabilities and permanent loss of function.^3,4^

Patients with HNC are often more frail than patients with other forms of solid malignancies.^5^ Frailty can be defined as being prone to adverse outcomes and declines in quality of life after a stressful event (eg, oncologic treatment) due to decreased physiologic reserves and homeostatic mechanisms.^6,7^

Locoregional tumor control and survival as primary outcomes are of high importance. However, especially in the older patients, a shift toward patient-centered outcomes is increasingly advocated, with an emphasis on quality of life and maintaining independence as guiding determinants in treatment decisions.^8^

A valuable patient-centered outcome is the amount of time spent at home, since most patients prefer that over time spent in hospital.^9,10^ With so many patients with HNC being frail, the risk of postoperative complications and acute radiation-induced toxicity is high,^5,11,12^ resulting in the need to spend more time in hospital. Although not a direct reflection of time spent at home, the number of hospital admission days can be used as alternative.^9,10^

In the Netherlands, HNC care is centralized into 8 head and neck oncology centers (HNOCs). Most HNOCs have implemented fast-track diagnostic workup trajectories. Consideration of patient-centered outcomes and shared decision making take time and can delay oncological workup. The effects of these delays can be serious due to tumor progression during the waiting time. This can result in more extensive treatment and lower survival rates.^13-15^

To ensure timely treatment initiation, quality indicator norms are set in some countries, such as Denmark and the Netherlands.^16,17^ In the latter, this norm is set at 30 days, starting from first consultation at the HNOC to start of treatment.^16^ However, this is achieved in only 34% of the patients diagnosed within the HNOC,^18^ underlining the need to identify predictors of delay and adjust care pathways accordingly. Internationally, a 30-day cutoff is frequently studied and pursued.^13,15,19^

The effect of delay in hospitalization in the year following treatment in patients with HNC is unclear. Furthermore, the effect of delay on overall survival and locoregional tumor control in the subgroup of older patients with HNC is not yet established. This multicenter study aims to investigate these associations in 2 high-volume tertiary referral centers to provide guidance in shared decision making in the current real-life population.

## Methods

### Study Design and Patient Selection

All consecutive patients with newly diagnosed head and neck squamous cell carcinoma (HNSCC) seen between 2015 and 2017 in the outpatients clinics of the University Medical Center Groningen (UMCG) and the Netherlands Cancer Institute (Antoni van Leeuwenhoek hospital, Amsterdam [AvL]) were included. Both hospitals are 1 of the 8 HNOCs within the centralized care setting for patients with HNSCC in the Netherlands, implemented by the Dutch Head and Neck Society.

For the UMCG, patients were prospectively enrolled in the OncoLifeS data biobank (Dutch Trial Register NL7839).^20^ For the AvL, patients were retrospectively included through a database management system.

To be eligible for inclusion, patients had to be ≥60 years old, presenting with first primary HNSCC in the oral cavity, oropharynx, hypopharynx, or larynx. Patients with distant metastasis or synchronous second primary tumors, patients who died before the start of treatment, and patients treated elsewhere were excluded.

The current study protocol was approved by the OncoLifeS scientific board (UMCG) and the Institutional Review Board (AvL). All cases were discussed in the local multidisciplinary tumor board and treated according to international guidelines.

### Definitions and Data Collection

The care pathway interval (CPI) was defined as the number of calendar days between the first visit in the HNOC and the start of treatment (ie, the first day of radiotherapy or chemoradiation or the day of surgery).^21^ CPI and all analysis involving CPI as a dependent or independent parameter were calculated for cases managed with curative intention. Based on internationally used cutoffs and the quality indicator norm set by the Dutch Head and Neck Society, CPI was dichotomized into patients starting treatment <30 days and ≥30 days (delayed group).^16^

Patient, tumor, and treatment characteristics were collected and supplemented with CPI and follow-up data. Tumor stage was reported with the UICC TNM classification (seventh edition; Union for International Cancer Control).^22^ The presence of comorbidities was graded with the ACE-27 (Adult Comorbidity Evaluation–27).^23^ Polypharmacy was defined as use of ≥5 medications.

The number of days spent in hospital (any department, excluding outpatient clinic visits) was measured in the first year after treatment initiation. For analyses, hospital admission days were dichotomized into ≤14 vs >14 days, as defined by Chesney et al.^9^

### Statistical Analysis

SPSS Statistics version 25.0 (IBM Corp) was used for analyses. Descriptive statistics were presented depending on their distribution, and comparisons were made via the Student *t* test, Mann-Whitney *U* test, or χ^2^ test.

The association between covariables and CPI (dichotomized <30 and ≥30 days) was analyzed with logistic regression analysis. Logistic regression was also used to assess predictors for >14 hospital admission days (hospitalization). Age was analyzed as a continuous and dichotomized value (<70 vs ≥70 years). All independent factors with *P* < .1 in univariable analysis were included in the multivariable analysis.

Cox regression analyses were performed to assess the effect of delay on 2-year overall survival and recurrence risk, establishing hazard ratios (HRs; >1 indicating a higher risk of dying or recurrence) after checking whether the Cox proportional hazard assumption was met. A 2-sided *P* < .05 was considered statistically significant.

## Results

### Patient Characteristics and Differences Between Centers

**Figure 1.**
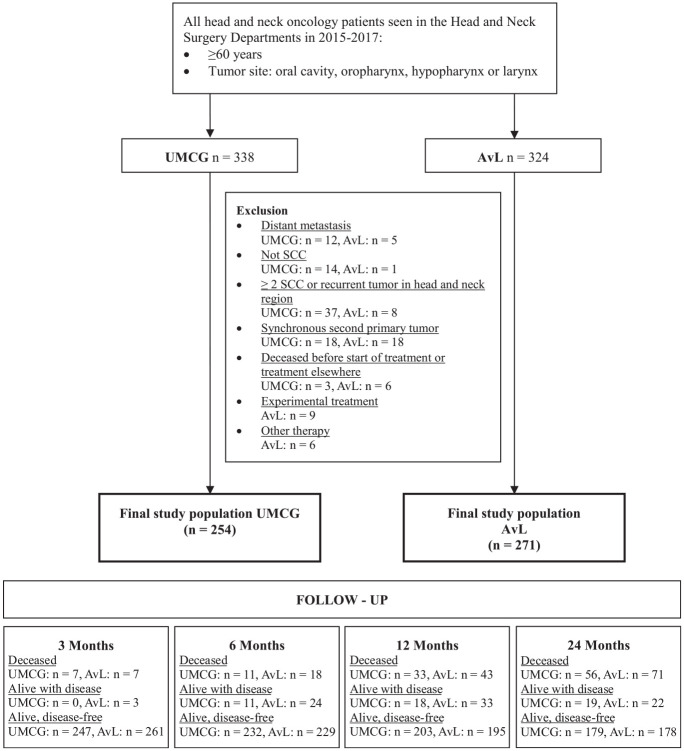
Flowchart of study population, including inclusion and exclusion criteria and follow-up characteristics. AvL, Antoni van Leeuwenhoek hospital (Amsterdam); SCC, squamous cell carcinoma; UMCG, University Medical Center Groningen.

In total, 525 patients were enrolled in this study (UMCG, n = 254; AvL, n = 271; **Figure 1**). The mean ± SD age was 70.7 ± 7.6 years and the majority were male (70.7%; **Table 1**). This did not statistically differ between centers, nor did smoking and drinking status, body mass index, and comorbidities.

**Table 1.** Baseline Characteristics of Study Population.^a^

Characteristic	All (N = 525)	UMCG (n = 254)	AvL (n = 271)	*P* value
Age, y				
Mean ± SD	70.7 ± 7.6	71.3 ± 7.4	70.3 ± 7.9	.132
Interquartile range	64.2-75.9	66.0-75.8	64.0-76.0	
Sex				.631
Male	371 (70.7)	182 (71.7)	189 (69.7)	
Female	154 (29.3)	72 (28.3)	82 (30.3)	
Smoking status				.302
Never	58 (12.2)	20 (9.9)	38 (14.0)	
Former	240 (50.6)	102 (50.2)	138 (50.9)	
Current	176 (37.1)	81 (39.9)	95 (35.1)	
Drinking status				.629
Never	103 (22.1)	47 (24.0)	56 (20.7)	
Former	73 (15.6)	33 (16.8)	40 (14.8)	
Mild/moderate	164 (35.1)	63 (32.1)	101 (37.3)	
Heavy	127 (27.2)	53 (27.0)	74 (27.3)	
ACE-27				.866
None	89 (17.6)	38 (16.2)	51 (18.8)	
Mild	185 (36.6)	89 (38.0)	96 (35.4)	
Moderate	148 (29.3)	68 (29.1)	80 (29.5)	
Severe	83 (16.4)	39 (16.7)	44 (16.2)	
Polypharmacy				**.001**
0 or <5 medications	272 (59.3)	130 (68.8)	142 (52.6)	
≥5 medications	187 (40.7)	59 (31.2)	128 (47.4)	
Body mass index				.180
Low	21 (4.6)	5 (2.6)	16 (6.1)	
Middle	211 (46.4)	87 (45.5)	124 (47.0)	
High	223 (49.0)	99 (51.8)	124 (47.0)	
Tumor site				**<.001**
Oral cavity	155 (29.5)	70 (27.6)	85 (31.4)	
Oropharynx	149 (28.4)	56 (22.0)	93 (34.3)	
Hypopharynx	39 (7.4)	16 (6.3)	23 (8.5)	
Larynx	182 (34.7)	112 (44.1)	70 (25.8)	
Stage of disease				**<.001**
I	120 (22.9)	77 (30.3)	43 (15.9)	
II	81 (15.4)	29 (11.4)	52 (19.2)	
III	86 (16.4)	44 (17.3)	42 (15.5)	
IV	238 (45.3)	104 (40.9)	134 (49.4)	
Treatment intention				**<.001**
Curative	482 (91.8)	245 (96.5)	237 (87.5)	
Palliative	43 (8.2)	9 (3.5)	34 (12.5)	
Curative treatment modality				**<.001**
Surgery	193 (40.0)	112 (45.7)	81 (34.2)	
Reconstructive	69 (35.8)	41 (36.6)	28 (34.6)	.123
Radiotherapy	173 (35.9)	92 (37.6)	81 (34.2)	
Chemoradiation	116 (24.1)	41 (16.7)	75 (31.6)	

Abbreviations: ACE-27, Adult Comorbidity Evaluation–27; AvL, Antoni van Leeuwenhoek hospital (Amsterdam); UMCG, University Medical Center Groningen.

a Values are presented as No. (%) unless noted otherwise. Bold indicates *P* < .05.

The proportion of patients with polypharmacy was larger in the UMCG (68.8% vs 52.6%, *P* = .001). Patients with oropharyngeal cancer and stage IV tumors were more frequently represented in the AvL group than the UMCG group (34.3% vs 22.0% [*P* < .001] and 49.4% vs 40.9% [*P* < .001], respectively). In the UMCG, the proportion of patients with laryngeal cancer was higher (44.1% vs 25.8%).

Most patients were treated with curative intention (91.8%). Surgery was the treatment modality most frequently reported (UMCG, 45.7%; AvL, 34.2%), while the proportion of patients treated with chemoradiation differed (UMCG, 16.7%; AvL, 31.6%).

### CPI and Determinants of Delay

**Figure 2.**
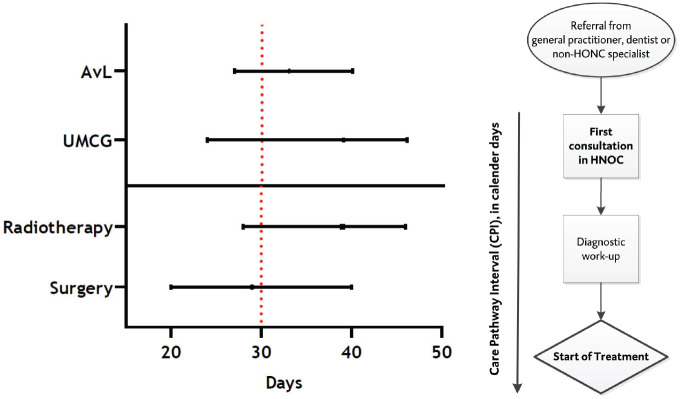
Details on the CPI (n = 482 patients with curative intention). CPI for AvL vs UMCG: *P* = .060. CPI for patients treated with radiotherapy vs surgery: *P* < .001. Dotted red line (30 days) represents the Dutch guideline. For AvL: median CPI for surgery was 34.0 (IQR, 27.0-43.0) as compared with 31.5 (IQR, 27.0-39.0) for initial radiotherapy (*P* = .375). For UMCG: median CPI for surgery was 26.5 (IQR, 15.8-36.0) as opposed to 40.0 (IQR, 39.0-53.0) for initial radiotherapy (*P* < .001). AvL, Antoni van Leeuwenhoek hospital (Amsterdam); CPI, care pathway interval; HNOC, head and neck oncology center; IQR, interquartile range; UMCG, University Medical Center Groningen.

The median interval between first consultation and start of treatment (CPI) was 39.0 days for the UMCG as compared with 33.0 for the AvL (*P* = .060; **Figure 2**). In total, 175 patients (36.3%) started treatment within 30 days (UMCG, 35.1%; AvL, 37.6%; *P* = .576). Patients treated with initial surgery had a median CPI of 29.0, as opposed to 39.0 days for patients with initial radiotherapy (*P* < .001).

In the univariable model, current smoking status, advanced-stage tumor at diagnosis, and initial treatment with radiotherapy or chemoradiation were associated with delay (CPI ≥30 days) in treatment initiation (**Table 2**). In the multivariable model, current smoking status (odds ratio [OR], 2.2 [95% CI, 1.1-4.6]; *P* = .026), stage IV tumors (OR, 3.1 [95% CI, 1.7-5.8]; *P* < .001), and initial radiotherapy (OR, 4.2 [95% CI, 2.4-7.2]; *P* > .001) remained significantly associated with delay.

**Table 2.** Univariable and Multivariable Logistic Regression Analyses for CPI ≥30 Calendar Days.^a^

	Univariable		Multivariable	
Variable	Odds ratio (95% CI)	*P* value	Odds ratio (95% CI)	*P* value
**Patient characteristics**				
Age: ≥70 y	0.89 (0.61-1.29)	.541		
Sex: female	0.93 (0.61-1.40)	.717		
Smoking status				
Never	Reference		Reference	
Former	1.01 (0.55-1.86)	.963	1.09 (0.56-2.13)	.799
Current	2.30 (1.20-4.41)	**.012**	2.24 (1.10-4.55)	**.026**
Drinking status				
Never	Reference			
Former	1.11 (0.58-2.11)	.761		
Mild/moderate	0.94 (0.56-1.60)	.829		
Heavy	1.62 (0.90-2.89)	.105		
Body mass index				
Low	Reference			
Middle	1.35 (0.50-3.64)	.559		
High	1.00 (0.37-2.67)	.995		
ACE-27				
None	Reference			
Mild	1.05 (0.62-1.79)	.862		
Moderate	0.97 (0.56-1.70)	.921		
Severe	1.09 (0.56-2.11)	.806		
Polypharmacy	1.20 (0.80-1.81)	.381		
**Tumor and treatment characteristics**				
Tumor site				
Oral cavity	Reference			
Oropharynx	1.34 (0.81-2.21)	.250		
Hypopharynx	1.03 (0.48-2.22)	.945		
Larynx	0.95 (0.60-1.50)	.815		
Stage of disease				
I	Reference		Reference	
II	2.52 (1.38-4.61)	**.003**	1.55 (0.78-3.09)	.211
III	1.86 (1.04-3.33)	**.036**	1.27 (0.62-2.61)	.510
IV	3.10 (1.94-4.97)	**<.001**	3.14 (1.70-5.80)	**<.001**
Treatment modality				
Surgery	Reference		Reference	
Radiotherapy	5.45 (3.34-8.89)	**<.001**	4.18 (2.43-7.18)	**<.001**
Chemoradiation	1.73 (1.08-2.77)	**.021**	0.77 (0.42-1.41)	.388
Center: AvL	0.90 (0.62-1.30)	.576		

Abbreviations: ACE-27, Adult Comorbidity Evaluation–27; AvL, Antoni van Leeuwenhoek hospital (Amsterdam); CPI, care pathway interval.

a Patients with curative treatment intention: n = 482. Bold indicates *P* < .1 for univariable and *P* < .05 for multivariable.

### Hospital Admission Days

**Figure 3.**
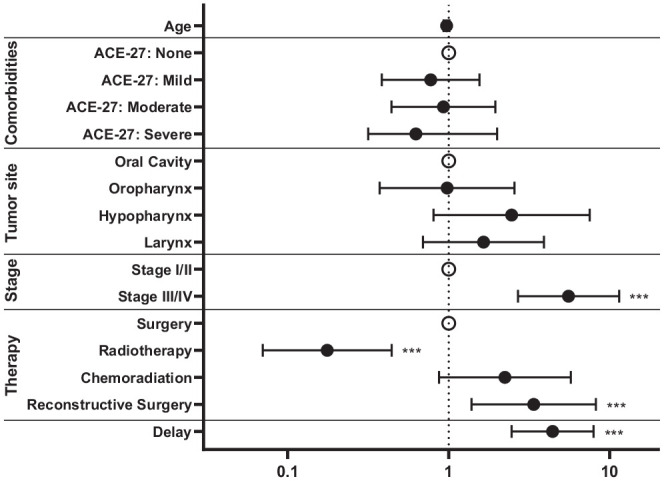
Forrest plot displaying the odds ratio for increased number of hospital admission days. Multivariable regression model for >14 hospital admission days in the year following start of treatment with curative intention (n = 482). Error bars indicate 95% CI. ***P < .001. ACE-27, Adult Comorbidity Evaluation–27.

The mean number of days spent in hospital in the first year after the start of curative treatment was 9.5 ± 13.6 for UMCG patients and 10.3 ± 15.7 for AvL patients (*P* = .096). Age, comorbidities, tumor site, stage, treatment modality, reconstructive surgery, and delay in treatment initiation were associated with >14 hospital admission days in the univariable model. Delay in treatment initiation was a strong significant predictor of >14 days spent in hospital in the first year after treatment in an adjusted model (OR, 4.3 [95% CI, 2.4-7.8]; *P* < .001; **Figure 3** and Supplementary Table S1, available online).

Initial treatment with radiotherapy was associated with decreased chance of >14 hospital admission days (OR, 0.2 [95% CI, 0.1-0.4]; *P* < .001), whereas advanced tumor stage increased the risk of >14 days spent in hospital (for stage IV tumors; OR, 9.9 [95% CI, 3.5-28.2]; *P* < .001). Reconstructive surgery was a significant predictor for longer hospital stay (>14 days) in the adjusted model as well (OR, 3.1 [95% CI, 1.3-7.7]; *P* = .015). Similar results were observed when time to treatment was analyzed as a continuous variable (OR, 1.1 per day [95% CI, 1.03-1.07]; *P* < .001; Supplemental Table S2, available online).

### Overall Survival and Recurrence

After 2 years, 127 patients were deceased (24.2%; **Figure 1**). Delay (CPI ≥30 days) was not associated with hazard of dying in univariable analysis (HR, 1.2 [95% CI, 0.8-1.9]; *P* = .285). Time to treatment as a continuous variable was also not associated with decreased survival in univariable analysis (HR, 1.0 [95% CI, 1.0-1.0]; *P* = .436). In the multivariable model, the following indicated an increased hazard of dying within 2 years after start of treatment: low BMI (HR, 3.4 [95% CI, 1.5-7.7]; *P* = .003), middle BMI (HR, 1.6 [95% CI, 1.0-2.6]; *P* = .034), oral cavity carcinomas (HR, 3.0 [95% CI, 1.6-5.7]; *P* = .001), and stage IV tumors (HR, 4.6 [95% CI, 2.0-10.7]; *P* < .001) (**Table 3**).

**Table 3.** Cox Regression Model Displaying the Hazard of Dying Within 2 Years After Start of Treatment With Curative Intention (n = 482).^a^

	Univariable		Multivariable	
Variable	Hazard ratio (95% CI)	*P* value	Hazard ratio (95% CI)	*P* value
**Delay**				
Cutoff: ≥30 d	1.24 (0.84-1.85)	.285		
Continuous	1.00 (0.99-1.01)	.436		
**Patient characteristics**				
Age: ≥70 y	1.75 (1.19-2.56)	**.004**	1.38 (0.90-2.14)	.142
Sex: female	1.43 (0.97-2.12)	**.073**	0.95 (0.57-1.56)	.824
Smoking status				
Never	Reference			
Former	1.11 (0.56-2.20)	.760		
Current	1.40 (0.70-2.79)	.338		
Drinking status				
Never	Reference		Reference	
Former	0.98 (0.52-1.83)	.973	1.44 (0.73-2.84)	.298
Mild/moderate	0.57 (0.32-1.01)	**.053**	0.76 (0.40-1.44)	.396
Heavy	1.17 (0.69-1.98)	.572	1.28 (0.71-2.32)	.416
Body mass index				
Low	3.36 (1.62-6.97)	**.001**	3.44 (1.54-7.70)	**.003**
Middle	1.58 (1.03-2.41)	**.035**	1.63 (1.04-2.57)	**.034**
High	Reference		Reference	
ACE-27				
None	Reference		Reference	
Mild	2.00 (1.00-4.01)	**.050**	1.66 (0.77-3.57)	.195
Moderate	2.74 (1.36-5.51)	**.005**	2.17 (0.98-4.80)	.057
Severe	3.18 (1.51-6.72)	**.002**	1.79 (0.71-4.50)	.217
Polypharmacy	1.73 (1.16-2.58)	**.008**	1.52 (0.96-2.42)	.076
**Tumor and treatment characteristics**				
Tumor site				
Oral cavity	3.16 (1.96-5.10)	**<.001**	2.96 (1.55-5.66)	**.001**
Oropharynx	1.92 (1.14-3.21)	**.014**	1.57 (0.78-3.14)	.206
Hypopharynx	2.27 (1.11-4.64)	**.024**	1.18 (0.45-3.06)	.737
Larynx	Reference		Reference	
Stage of disease				
I	Reference		Reference	
II	2.12 (0.99-4.52)	**.053**	2.38 (0.94-6.04)	.068
III	1.45 (0.64-3.29)	.371	1.05 (0.34-3.22)	.939
IV	4.10 (2.22-7.54)	**<.001**	4.63 (2.01-10.67)	**<.001**
Treatment modality				
Surgery	Reference			
Radiotherapy	1.08 (0.70-1.64)	.738		
Chemoradiation	0.95 (0.58-1.54)	.830		
Reconstructive surgery	1.47 (0.84-2.57)	.174		
Center: AvL	1.07 (0.74-1.55)	.727		

Abbreviations: ACE-27, Adult Comorbidity Evaluation–27; AvL, Antoni van Leeuwenhoek hospital (Amsterdam).

a Bold indicates *P* < .1 for univariable and *P* < .05 for multivariable.

In univariable analysis, delay as a continuous variable (per day) was associated with hazard of recurrence; however, this association did not remain significant in the adjusted model. Age ≥70 years (HR, 1.8 [95% CI, 1.2-2.8]; *P* = .005), former drinking status (HR, 2.2 [95% CI, 1.1-4.4]; *P* = .020), heavy drinking status (HR, 2.4 [95% CI, 1.3-4.5]; *P* = .004), and stage IV tumors (HR, 3.4 [95% CI, 1.7-6.7]; *P* = .001) resulted in a significantly increased hazard of recurrent disease within 2 years after treatment initiation in a multivariable model (Supplemental Table S3, available online).

## Discussion

In this multicenter cohort study, the effect of delay on hospitalization, overall survival, and recurrence risk in older patients with HNC was investigated. Treatment was initiated within 30 days after first consultation in only about one-third of the cases (36.3%). Patients treated with definitive radiotherapy had a significant, 5-times higher risk to delayed treatment initiation as compared with patients treated with initial surgery.

Delay was an independent predictor for hospitalization (adjusted for age, comorbidities, tumor site and stage, and treatment modality), highlighting the importance of timely treatment. Advanced tumor stage was associated with hospitalization as well, whereas patients treated with radiotherapy were likely to experience fewer days in hospital in the year posttreatment as compared with patients treated with surgery.

Delay in treatment initiation was not associated with overall survival or tumor recurrence.

### CPI and Determinants of Delay

The proportion of patients starting treatment within 30 days was similar in both centers. Because of the centralized setting of HNC care in the Netherlands and the similar treatment protocols according to national guidelines, the 2 high-volume HNOCs were highly comparable. This study confirms the difficulties encountered in aiming for early start of treatment while focusing on patient-centered outcomes and pursuing shared decision making at the same time.

In this study, we did not find an association between delay and age. The proportion of patients treated within 30 days is comparable to other studies describing younger patients^21^ or investigating delay in elderly patients (<70 vs ≥70 years).^24^ This can be explained by the fact that due to the lifestyle of patients with HNC, a mismatch between chronological and biological age can often be experienced.^5^ Although no consensus exists regarding the use of an age cutoff, this study used a lower cutoff (60 years vs 70) to not miss possibly younger frail patients.

The association between current smoking status and delay is somewhat surprising. This association has not been extensively described, although the 3 reports that did study this association did not find a significant contribution to delay.^25-27^ An older report examined predictors for delay in first presentation with HNC and did find heavy drinkers and smokers to be associated with delay. The authors suggested that dismissive behavior and underestimating the severity of illness might be the underlying explanation, presuming patient delay rather than professional delay.^28^

Stage IV tumors and radiotherapy are predictors for delay, corresponding to existing literature.^13,18,21,29-31^ Radiotherapy treatment requires extensive pretreatment planning (dental assessment and extractions, molds, and mask preparations). Furthermore, advanced-stage tumors might be eligible for radiotherapy treatment, whereas lower-stage tumors can be surgically managed.

### Hospital Admission Days

The number of hospital admission days is frequently used as a measure of the amount of time spent at home.^9,10^ Even though most patients highly value their independence and time at home,^32,33^ studies assessing the time at home of older patients with HNC after treatment are scarce, and the effect of delay on hospital admission days has not been previously investigated.

This study found that patients with a delay (≥30 days) do have a 4-times higher risk of hospitalization (>14 hospital admission days) in the year after treatment initiation. This analysis is adjusted for confounders (ie, age, comorbidities, tumor site and stage, and treatment modality including major surgery), given that surgically treated patients generally start their treatment earlier. For postoperative patients, loss of time at home is associated with poor functional outcomes (depression, difficulty with self-care, limited social activity, and mobility).^34^

Although the consequences of decreased time at home are alarming, the explanation for this finding in the elderly population is less obvious. Prolonged time-to-treatment initiation might result in tumor progression^35^ and more extensive (surgical) treatment and subsequent longer in-hospital recovery, although this association cannot be objectively determined retrospectively. Tests to rule out collinearity of covariables were performed, confirming an insignificant collinearity among the variables. Even though the health insurance policies and supportive care at home facilities are equal for all inhabitants in the Netherlands, these findings should be interpreted with caution, since it is difficult to adjust for possible socioeconomic drivers of prolonged hospital stay.

These findings should be taken into consideration during pretreatment counseling and can be used to manage patients’ expectations toward hospitalization time. Also, posttreatment decline in functioning needs to be addressed during counseling at the outpatient clinic. Implementation of early geriatric assessment in the early diagnostic phase may assist in personalized pretreatment counseling.^10,36^ Our results should be interpreted in the absence of preoperative geriatric assessment, since frailty is a known predictor of hospitalization.^37^

Advanced tumor stages were associated with hospitalization, which might be explained by the more sophisticated and multidisciplinary surgical treatments for these selected patients (ie, collaboration with the plastic surgeon, higher risk of postoperative infection after extensive surgery^38^).

### Overall Survival and Recurrence Rate

A CPI ≥30 days was not associated with overall survival or recurrence rate. Other studies, in contrast, did find an association between delay and lower overall survival rates, although this effect was significant only for delays of 45 to 90 days.^13,31,39^ In this cohort, the number of patients with such extensive delays was too small for analysis.

This illustrates the complicated interpretation of previous studies. First, the definition used for “delay” is not often well defined and widely varies among reports. Second, no consensus exists on the number of days regarded as an acceptable waiting time. A recent systematic review on this topic showed a wide range in median CPI of 20 to 55.5 days.^14^

Rygalski et al reported a median time to surgery of 33 days for a large cohort of patients with HNC (n = 37,730), starting from date of diagnosis (either clinical description or histologically confirmed). Longer time to surgery was associated with decreased overall survival; however, this effect was apparent only after time to surgery >67 days, far above the study’s median^40^ and the median in our study.

The effect of delay on locoregional tumor control is less extensively analyzed, and the reported findings show conflicting results. In line with our findings, 2 studies did not find a significant association between longer waiting time and recurrence.^26,27^ Liao et al, however, described an increased risk of recurrence of patients with a waiting time >60 days.^41^

In conclusion, the effect of delay in time-to-treatment initiation on overall survival and recurrence risk seems most prominent in delays >60 days—a situation that rarely occurs in the setting of centralized HNC treatment in the Netherlands.

### Strengths and Limitations

The data used in this study and the criteria for inclusion and exclusion represent real-world data, with minimal risk of selection bias. Patients with recurrent disease or synchronous secondary HNSCC were excluded because they have increased chances of worse outcome regarding the primary endpoints analyzed in this study (hospital admission days and overall survival) as a result of a difference in treatment options (ie, previous irradiation in case of recurrent disease or more extensive treatment in case of multiple primary tumors). Moreover, these patients will enter a different care pathway as compared with patients with first primary HNSCC, having a known general health status (recurrence) or a need for additional investigations (second primary tumors).

A longer follow-up on days at home could add to the literature. In line with previous studies, when time at home is mentioned, the reverse is actually measured: the time spent in hospital.^9,10^ It cannot be stated with certainty that patients were actually at home; therefore, time spent out of hospital might be a more accurate terminology.

Using cutoffs for delay and hospitalization—variables with a skewed nature—can be arbitrary. As such, dichotomization might be the most sensible approach. A disadvantage of this method can be that a shift in the number of patients per group results in different outcome. To minimize the impact on the results and subsequent conclusions, analyses on the same variables based on linear procedures with continuous dependent variables were performed and did not lead to significant alterations (Supplementary Information, available online).

The association of specific surgical procedures or radiotherapy treatment on hospitalization time was not analyzed in this study; however, the intention was to establish a pragmatic impression of the time spent at home after HNC treatment.

## Conclusion

This study highlights the importance and challenges to ensure timely treatment initiation of HNC. A prolonged CPI (≥30 days) was an independent predictor of hospitalization in older patients with HNC during the year following treatment. In the present study, delay in treatment initiation was not associated with decreased overall survival or recurrence risk.

During oncologic workup, taking time to consider patient-centered outcomes (including minimizing time spent in hospital) while ensuring timely start of treatment requires well-structured, fast-track care pathways.

## Supplemental Material

sj-docx-1-oto-10.1177_01945998211072828 – Supplemental material for Impact of Delay on Hospitalization in Older Patients With Head and Neck Cancer: A Multicenter Study Click here for additional data file. Supplemental material, sj-docx-1-oto-10.1177_01945998211072828 for Impact of Delay on Hospitalization in Older Patients With Head and Neck Cancer: A Multicenter Study by Rosanne C. Schoonbeek, Suzanne Festen, Roza Rashid, Boukje A.C. van Dijk, György B. Halmos and Lilly-Ann van der Velden in Otolaryngology–Head and Neck Surgery
