# Supplementary material for: Impact of Delay on Hospitalization in Older Patients With Head and Neck Cancer: A Multicenter Study
Source: Otolaryngol Head Neck Surg. 2022 Jan 19;167(4):678–87. doi: 10.1177/01945998211072828 (PMC9527368; doi:10.1177/01945998211072828)
Supplement: sj-docx-1-oto-10.1177_01945998211072828 – Supplemental material for Impact of Delay on Hospitalization in Older Patients With Head and Neck Cancer: A Multicenter Study [file sj-docx-1-oto-10.1177_01945998211072828.docx]

**Supplementary Information**

**Supplementary Table 1^a^.** Uni- and multivariable logistic regression analysis for hospital admission >14 days during the first year after start of curative intention treatment (n = 482). ^a^*Corresponding to Figure 3.*

|  | **Univariable** | | **Multivariable** | |
| --- | --- | --- | --- | --- |
| **Variable** | **Odds Ratio (95% CI)** | **p - value** | **Odds Ratio (95% CI)** | **p - value** |
| Delay (cut-off ≥30 days) | 2.73 (1.71 to 4.36) | **<0.001** | 4.31 (2.39 to 7.80) | **<0.001** |
| **Patient Characteristics** |  |  |  |  |
| Age (continuous) | 0.95 (0.92 to 0.98) | **<0.001** | 0.97 (0.93 to 1.01) | 0.127 |
| Age (≥70y) | 0.47 (0.31 to 0.71) | **<0.001** |  |  |
| Sex (female) | 1.13 (0.72 to 1.75) | 0.600 |  |  |
| Smoking status |  |  |  |  |
| never | ref | ref |  |  |
| former | 1.16 (0.58 to 2.31) | 0.677 |  |  |
| current | 1.32 (0.65 to 2.69) | 0.446 |  |  |
| Drinking status |  |  |  |  |
| never | ref | ref |  |  |
| former | 1.22 (0.59 to 2.52) | 0.596 |  |  |
| mild/moderate | 1.45 (0.80 to 2.64) | 0.220 |  |  |
| heavy | 1.46 (0.78 to 2.74) | 0.237 |  |  |
| BMI |  |  |  |  |
| low | ref | ref |  |  |
| middle | 2.14 (0.60 to 7.69) | 0.242 |  |  |
| high | 1.83 (0.51 to 6.55) | 0.355 |  |  |
| ACE-27 |  |  |  |  |
| none | ref | ref | ref | ref |
| mild | 0.60 (0.34 to 1.03) | **0.065** | 0.79 (0.39 to 1.59) | 0.505 |
| moderate | 0.66 (0.37 to 1.17) | 0.156 | 0.92 (0.44 to 1.93) | 0.822 |
| severe | 0.52 (0.26 to 1.06) | **0.071** | 0.81 (0.32 to 2.04) | 0.655 |
| Polypharmacy | 0.87 (0.56 to 1.35) | 0.530 |  |  |
| **Tumor and treatment characteristics** |  |  |  |  |
| Tumor site |  |  |  |  |
| Oral cavity | ref | ref | ref | ref |
| Oropharynx | 1.15 (0.69 to 1.91) | 0.599 | 1.10 (0.40 to 2.99) | 0.860 |
| Hypopharynx | 2.43 (1.14 to 5.18) | **0.022** | 2.73 (0.87 to 8.57) | 0.086 |
| Larynx | 0.44 (0.26 to 0.76) | **0.003** | 1.91 (0.78 to 4.68) | 0.158 |
| Stage of disease |  |  |  |  |
| Stage I | ref | ref | ref | ref |
| Stage II | 2.53 (0.86 to 7.42) | **0.091** | 2.84 (0.87 to 9.24) | 0.084 |
| Stage III | 8.37 (3.23 to 21.68) | **<0.001** | 8.85 (2.98 to 26.32) | **<0.001** |
| Stage IV | 15.39 (6.48 to 36.57) | **<0.001** | 9.91 (3.48 to 28.24) | **<0.001** |
| Treatment modality |  |  |  |  |
| Surgery | ref | ref | ref | ref |
| Radiotherapy | 0.28 (0.16 to 0.52) | **<0.001** | 0.15 (0.06 to 0.38) | **<0.001** |
| Chemoradiation | 3.68 (2.26 to 5.98) | **<0.001** | 1.91 (0.73 to 5.00) | 0.187 |
| Major (reconstructive) surgery | 3.55 (2.10 to 6.00) | **<0.001** | 3.10 (1.25 to 7.69) | **0.015** |
| Center (AvL) | 1.32 (0.88 to 1.97) | 0.179 |  |  |

**Supplementary Table 2.** Uni- and multivariable logistic regression analysis for hospital admission >14 days during the first year after start of treatment with curative intention (n = 482, using delay as continuous variable).

|  | **Univariable** | | **Multivariable** | |
| --- | --- | --- | --- | --- |
| **Variable** | **Odds Ratio (95% CI)** | **p - value** | **Odds Ratio (95% CI)** | **p - value** |
| Delay (continuous) | 1.03 (1.02 to 1.05) | **<0.001** | 1.05 (1.03 to 1.07) | **<0.001** |
| **Patient Characteristics** |  |  |  |  |
| Age (continuous) | 0.95 (0.92 to 0.98) | **<0.001** | 0.96 (0.92 to 1.00) | 0.062 |
| Age (≥70y) | 0.47 (0.31 to 0.71) | **<0.001** |  |  |
| Sex (female) | 1.13 (0.72 to 1.75) | 0.600 |  |  |
| Smoking status |  |  |  |  |
| never | ref | ref |  |  |
| former | 1.16 (0.58 to 2.31) | 0.677 |  |  |
| current | 1.32 (0.65 to 2.69) | 0.446 |  |  |
| Drinking status |  |  |  |  |
| never | ref | ref |  |  |
| former | 1.22 (0.59 to 2.52) | 0.596 |  |  |
| mild/moderate | 1.45 (0.80 to 2.64) | 0.220 |  |  |
| heavy | 1.46 (0.78 to 2.74) | 0.237 |  |  |
| BMI |  |  |  |  |
| low | ref | ref |  |  |
| middle | 2.14 (0.60 to 7.69) | 0.242 |  |  |
| high | 1.83 (0.51 to 6.55) | 0.355 |  |  |
| ACE-27 |  |  |  |  |
| none | ref | ref | ref | ref |
| mild | 0.60 (0.34 to 1.03) | **0.065** | 0.80 (0.40 to 1.61) | 0.526 |
| moderate | 0.66 (0.37 to 1.17) | 0.156 | 0.86 (0.41 to 1.81) | 0.692 |
| severe | 0.52 (0.26 to 1.06) | **0.071** | 0.82 (0.32 to 2.07) | 0.672 |
| Polypharmacy | 0.87 (0.56 to 1.35) | 0.530 |  |  |
| **Tumor and treatment characteristics** |  |  |  |  |
| Tumor site |  |  |  |  |
| Oral cavity | ref | ref | ref | ref |
| Oropharynx | 1.15 (0.69 to 1.91) | 0.599 | 1.06 (0.39 to 2.89) | 0.910 |
| Hypopharynx | 2.43 (1.14 to 5.18) | **0.022** | 2.53 (0.80 to 7.97) | 0.112 |
| Larynx | 0.44 (0.26 to 0.76) | **0.003** | 1.74 (0.70 to 4.29) | 0.231 |
| Stage of disease |  |  |  |  |
| Stage I | ref | ref | ref | ref |
| Stage II | 2.53 (0.86 to 7.42) | **0.091** | 2.40 (0.73 to 7.85) | 0.148 |
| Stage III | 8.37 (3.23 to 21.68) | **<0.001** | 7.86 (2.63 to 23.48) | **<0.001** |
| Stage IV | 15.39 (6.48 to 36.57) | **<0.001** | 8.33 (2.93 to 23.67) | **<0.001** |
| Treatment modality |  |  |  |  |
| Surgery | ref | ref | ref | ref |
| Radiotherapy | 0.28 (0.16 to 0.52) | **<0.001** | 0.14 (0.05 to 0.38) | **<0.001** |
| Chemoradiation | 3.68 (2.26 to 5.98) | **<0.001** | 1.87 (0.71 to 4.93) | 0.204 |
| Major (reconstructive) surgery | 3.55 (2.10 to 6.00) | **<0.001** | 3.09 (1.25 to 7.62) | **0.014** |
| Center (AvL) | 1.32 (0.88 to 1.97) | 0.179 |  |  |

**Supplementary Table 3.** Cox regression model displaying the hazard of recurrence within two years after start of treatment with curative intention (n = 482).

|  | **Univariable** | | **Multivariable** | |
| --- | --- | --- | --- | --- |
| **Variable** | **Hazard Risk (95% CI)** | **p - value** | **Hazard Risk (95% CI)** | **p - value** |
| Delay (cut-off ≥30 days) | 1.24 (0.83 to 1.85) | 0.299 |  |  |
| Delay (continuous) | 1.01 (1.00 to 1.02) | **0.019** | 1.01 (1.00 to 1.02) | 0.277 |
| **Patient Characteristics** |  |  |  |  |
| Age (continuous) | 1.01 (0.99 to 1.04) | 0.392 |  |  |
| Age (≥70y) | 1.56 (1.07 to 2.83) | **0.022** | 1.81 (1.20 to 2.75) | **0.005** |
| Sex (female) | 1.08 (0.72 to 1.64) | 0.702 |  |  |
| Smoking status |  |  |  |  |
| never | ref | ref |  |  |
| former | 0.84 (0.46 to 1.55) | 0.572 |  |  |
| current | 1.01 (0.55 to 1.89) | 0.964 |  |  |
| Drinking status |  |  |  |  |
| never | ref | ref | ref | ref |
| former | 1.80 (0.93 to 3.47) | **0.080** | 2.22 (1.13 to 4.35) | **0.020** |
| mild/moderate | 1.12 (0.61 to 2.07) | 0.709 | 1.46 (0.78 to 2.74) | 0.236 |
| heavy | 1.98 (1.10 to 3.58) | **0.023** | 2.43 (1.32 to 4.47) | **0.004** |
| BMI |  |  |  |  |
| low | ref | ref |  |  |
| middle | 1.27 (0.46 to 3.50) | 0.651 |  |  |
| high | 0.97 (0.35 to 2.70) | 0.956 |  |  |
| ACE-27 |  |  |  |  |
| none | ref | ref | ref | ref |
| mild | 1.17 (0.66 to 2.10) | 0.591 | 1.10 (0.59 to 2.05) | 0.770 |
| moderate | 1.67 (0.93 to 3.00) | **0.085** | 1.38 (0.74 to 2.57) | 0.316 |
| severe | 1.30 (0.65 to 2.59) | 0.465 | 1.12 (0.54 to 2.35) | 0.759 |
| Polypharmacy | 1.15 (0.77 to 1.72) | 0.482 |  |  |
| **Tumor and treatment characteristics** |  |  |  |  |
| Tumor site |  |  |  |  |
| Oral cavity | 2.24 (1.38 to 3.64) | **0.001** | 1.53 (0.90 to 2.61) | 0.119 |
| Oropharynx | 1.68 (1.02 to 2.79) | **0.043** | 0.81 (0.44 to 1.47) | 0.482 |
| Hypopharynx | 1.57 (0.72 to 3.46) | 0.261 | 0.50 (0.19 to 1.30) | 0.154 |
| Larynx | ref | ref | ref | ref |
| Stage of disease |  |  |  |  |
| Stage I | ref | ref | ref | ref |
| Stage II | 1.79 (0.84 to 3.81) | 0.131 | 1.20 (0.54 to 2.64) | 0.653 |
| Stage III | 1.60 (0.74 to 3.46) | 0.229 | 1.57 (0.69 to 3.60) | 0.285 |
| Stage IV | 3.52 (1.95 to 6.37) | **<0.001** | 3.35 (1.68 to 6.67) | **0.001** |
| Treatment modality |  |  |  |  |
| Surgery | ref | ref |  |  |
| Radiotherapy | 0.95 (0.61 to 1.48) | 0.830 |  |  |
| Chemoradiation | 1.17 (0.73 to 1.86) | 0.519 |  |  |
| Center (AvL) | 1.38 (0.95 to 2.01) | **0.091** | 1.38 (0.92 to 2.09) | 0.123 |
